# A Comparison of Clinical Outcomes Among Undenatured Collagen Type II, Glucosamine Sulfate, and Diacerein on Osteoarthritic Knee Treatment: A Randomized Clinical Trial

**DOI:** 10.2106/JBJS.OA.26.00225

**Published:** 2026-09-09

**Authors:** Rapeepat Narkbunnam, Kit Awirotananon, Keerati Chareancholvanich, Chaiwat Achawakulthep, Nath Adulkasem, Swist Chatmaitri, Paphon Hirunyachoke, Chaturong Pornrattanamaneewong

**Affiliations:** 1 Department of Orthopaedic Surgery, Faculty of Medicine Siriraj Hospital, Mahidol University, Bangkok, Thailand; 2 Department of Orthopaedic Surgery, Ratchaphiphat Hospital, Bangkok, Thailand; 3 Department of Orthopaedic Surgery, Nakhonpathom Hospital, Nakhon Pathom, Thailand

## Abstract

**Background:**

Knee osteoarthritis (OA) causes significant disability with limited effective treatments. Undenatured collagen type II (UC-II), prescription-grade crystalline glucosamine sulfate (pCGS), and diacerein have been proposed as alternatives to conventional analgesics, but their comparative efficacy remains uncertain. We hypothesized that each supplement would demonstrate superior efficacy over placebo in reducing total Western Ontario and McMaster Universities Arthritis Index (WOMAC) score at Week 24 in patients with mild-to-moderate knee OA.

**Methods:**

This 4-arm, randomized, double-blind, placebo-controlled trial enrolled 216 Thai adults with symptomatic knee OA (Kellgren-Lawrence grade 2-3). Participants received UC-II (40 mg once daily), pCGS (500 mg 3 times daily), diacerein (50 mg once to twice daily), or placebo for 24 weeks, with follow-up to 36 weeks. The primary outcome was total WOMAC score (22 items; pain, stiffness, and physical function; scored 0-220, higher = worse) at Week 24. Analyses followed intention-to-treat principles using multiple imputation by chained equations (m = 10) and linear mixed-effects models.

**Results:**

Of 216 randomized Thai participants, 208 (96.3%) completed follow-up (mean age 61.7 ± 7.1 years; 90.9% female; 73.6% Kellgren-Lawrence grade 2). At Week 24, no supplement demonstrated superiority over placebo. Between-group differences in total WOMAC score were as follows: UC-II −4.7 (95% CI −18.6 to 9.1), pCGS −8.1 (95% CI −21.8 to 5.7), and diacerein −12.3 (95% CI −25.9 to 1.3). All 95% CIs included zero, and all point estimates were at or below, and none clearly exceeded, the minimum clinically important difference threshold. At 36 weeks, results were consistent: UC-II −1.8 (95% CI −15.5 to 12.0), pCGS −4.8 (95% CI −18.4 to 8.7), and diacerein −1.3 (95% CI −14.9 to 12.3). Secondary outcomes including pain scores, joint space width, and functional performance showed no significant between-group differences. Adverse events were mild and comparable across groups; no serious adverse events occurred.

**Conclusions:**

Among Thai patients with mild-to-moderate knee OA, UC-II, pCGS, and diacerein did not demonstrate superiority over placebo in reducing pain or improving function at 24 weeks. These findings do not support their routine use as monotherapy for knee OA.

**Trial Registration:**

Thailand Clinical Trial Registry (TCTR20210901003).

**Level of Evidence:**

Therapeutic Level I. See Instructions for Authors for a complete description of levels of evidence.

## Introduction

Knee osteoarthritis (OA) is a leading cause of disability in older adults^1,2^, causing chronic pain, reduced mobility, and high healthcare costs^1^. Conventional treatments (NSAIDs, opioids, and acetaminophen) show limited, uncertain efficacy for knee OA pain relief^3,4^, and prolonged use carries gastrointestinal, renal, and cardiovascular risks^5^.

This has prompted interest in symptomatic slow-acting drugs for osteoarthritis^6^. Prescription-grade crystalline glucosamine sulfate (pCGS) and diacerein are proposed adjuncts to conventional therapy^2,6-9^. However, clinical evidence for these supplements remains inconclusive^2,10^, leading many guidelines to withhold endorsement^2,11-14^.

Recently, undenatured collagen type II (UC-II) has attracted attention for its novel mechanism in knee OA treatment^15,16^. Unlike other supplements, UC-II works by desensitizing T-cell–mediated immune responses toward articular collagen through oral tolerance, potentially reducing inflammatory responses that contribute to cartilage degradation^15,17,18^. Despite its theoretical promise, clinical evidence for UC-II remains limited^16^. The aim of this study was to compare the efficacy of pCGS, diacerein, and UC-II against placebo in improving knee function, physical performance, pain control, joint space preservation, and reducing the need for additional analgesics, while monitoring for complications.

## Materials and Methods

### Study Design

**Fig. 1:**
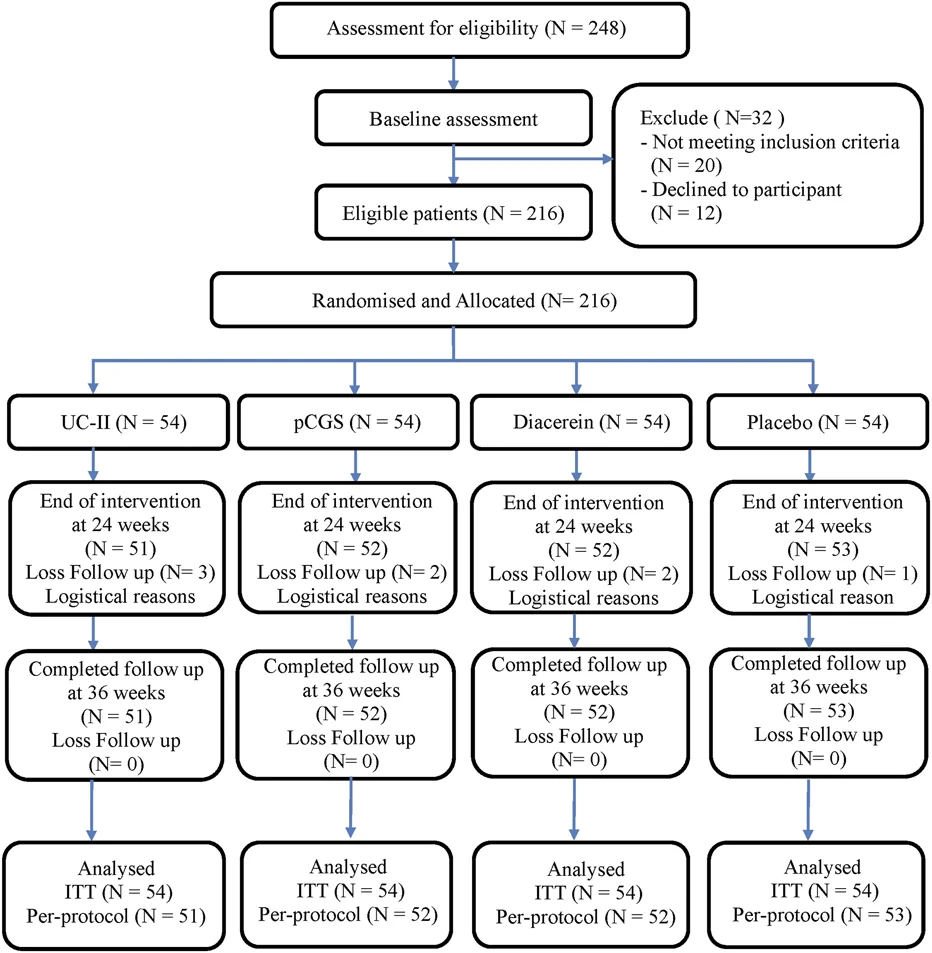
CONSORT flow diagram. Enrollment, randomization, and follow-up of participants across the 4 treatment arms (UC-II, pCGS, diacerein, and placebo) over 36 weeks. Of 216 randomized participants, 208 (96.3%) completed the study. Eight participants (3.7%) were lost to follow-up for logistical/nonmedical reasons and were included in the primary intention-to-treat analysis through multiple imputation by chained equations (m = 10). CONSORT = Consolidated Standards of Reporting Trials, ITT = intention-to-treat, pCGS = prescription-grade crystalline glucosamine sulfate, and UC-II = undenatured type II collagen.

This 4-arm, randomized, double-blind, placebo-controlled trial (TCTR20210901003; IRB COA 550/2021) enrolled 216 Thai adults with mild-to-moderate knee OA between July 2021 and March 2023. Participants were randomized 1:1:1:1 to UC-II, pCGS, diacerein, or placebo for 24 weeks, with follow-up to 36 weeks. Assessments were performed at baseline and Weeks 4, 12, 24, and 36. All provided written informed consent per the Declaration of Helsinki^19^. The study flow is shown in Figure 1.

### Participants

Participants aged 50 to 80 years with symptomatic, radiologically confirmed knee OA (Kellgren-Lawrence grade 2-3)^20^ meeting American College of Rheumatology/Arthritis Foundation (ACR) criteria^21^, Numeric Pain Rating Scale (NPRS) > 4, and no prior study supplements within 6 months were enrolled. Exclusion criteria included prior knee replacement; recent intra-articular corticosteroid (≤2 months) or hyaluronic acid (≤6 months); significant gastrointestinal, renal (GFR < 30 ml/min/1.73 m^2^), or hepatic disease; uncontrolled diabetes (HbA1c > 7.0%); allergy to study medications; recent cardiovascular or cerebrovascular disease; concurrent warfarin or aspirin; or impaired mobility. Participants with missing data on follow-up visits were included in the primary intention-to-treat (ITT) analysis; participants with poor adherence or missing data on follow-up visits performed according to the protocol were excluded from the per-protocol sensitivity analysis only.

### Randomization and Blinding

Computer-generated block randomization (block sizes 8 and 12) was performed by an independent statistician uninvolved in recruitment or assessment. Randomization codes were sealed in opaque envelopes, accessible only in emergencies. Supplements were repackaged into identical capsules by a nonaffiliated pharmacist in sequentially numbered, light-resistant packages. Participants, investigators, assessors, and analysts remained blinded throughout.

### Study Intervention

All participants followed an identical 3-times-daily oral regimen: UC-II 40 mg once daily (morning) with placebo at lunch/dinner; pCGS 500 mg 3 times daily; diacerein 50 mg once daily for month 1, then 50 mg twice daily (morning/evening) with midday placebo for the remaining 5 months; and placebo 3 times daily for controls. All participants also followed standardized lifestyle modification: quadriceps strengthening, range-of-motion exercises, low-impact aerobic activity (30 min/day), and avoidance of extreme knee flexion.

### Outcomes

All outcomes were assessed by blinded evaluators at baseline and Weeks 4, 12, 24, and 36. The primary outcome was the total Western Ontario and McMaster Universities Arthritis Index (WOMAC) score, using the validated Thai version^22^ (22 items assessing pain, stiffness, and physical function; scored 0-220, where higher scores indicate greater symptom burden)^23^. The primary timepoint was Week 24 (end of treatment); Week 36 was a prespecified secondary timepoint to assess carry-over effects following treatment discontinuation.

Secondary outcomes included pain intensity by the NPRS (0-10), radiographic joint space width (JSW) by standardized semiflexed standing radiography (20° flexion, 10° external rotation)^24^, physical performance by the 6-minute walk test (6MWT) and timed up and go (TUG) test^25-27^, and rescue analgesic consumption tracked via medication package verification. JSW inter-rater and intra-rater reliability were excellent to good (intraclass correlation coefficient 0.918-0.972 and 0.883-0.946, respectively; MDC_95_ 0.86-1.22 mm; Supplementary Table 1); JSW is designated exploratory given the short study duration. All secondary outcomes are exploratory; no multiplicity adjustment was performed. Adverse events were recorded at all visits and classified by type, severity, and relationship to study drug.

### Treatment Compliance Monitoring

Compliance was monitored via medication possession ratio (MPR), calculated by a blinded pharmacist from returned packages. Participants with MPR ≥80% were considered compliant; those below were retained in the primary ITT analysis but excluded from the per-protocol sensitivity analysis. All participants maintained daily medication diaries, with reminder contacts and pharmacist counseling provided as needed.

### Statistical Analyses

#### Sample Size Calculation

Sample size was calculated using nQuery Advisor (1-Way analysis of variance) based on Lugo et al. (2016)^17^, which used WOMAC VA3.1 (0-2,400 scale). Group means from that study yielded a scale-independent effect size Δ^2^ = 0.0812 (V = 6,828.5; σ = 290). With alpha = 0.05, power = 90%, and 4 arms, 45 participants per group was required. Accounting for an anticipated attrition rate of approximately 15% (n/(1−0.15) = 52.94), a target enrollment of 54 participants per group (N = 216) was set. Observed attrition in this trial (8/216, 3.7%) was substantially lower than anticipated.

#### Primary Analysis

All analyses followed ITT principles including all 216 participants. Missing data (n = 8; logistical reasons unrelated to treatment) were handled by multiple imputation by chained equations (Stata mi impute chained, m = 10; Bayesian linear regression imputation for continuous variables) under the missing-at-random assumption. The imputation model included treatment allocation and all WOMAC assessments (baseline, Weeks 4, 12, 24, and 36) to preserve the relationships among repeated measurements; estimates from the 10 imputed data sets were pooled using Rubin's rules. Imputed data sets were subsequently analyzed using linear mixed-effects models with treatment group, visit, group-by-visit interaction, and baseline WOMAC as covariates. The primary timepoint was Week 24; Week 36 was a prespecified secondary timepoint. The primary estimand was defined as the adjusted between-group difference in total WOMAC score at Week 24 among all randomized participants (treatment-policy strategy for intercurrent events), estimated as marginal means from the linear mixed-effects model described above. As the primary objective was to compare each active supplement against placebo independently, no formal adjustment for multiplicity was applied across the 3 primary treatment vs. placebo comparisons; results should be interpreted with this in mind. The results are presented as estimated marginal means and between-group differences with 95% CI; statistical significance was inferred from CI exclusion of zero (Consolidated Standards of Reporting Trials 2010).

#### Sensitivity Analyses

Prespecified sensitivity analyses included per-protocol analysis (MPR ≥80%), analysis of covariance adjusting for age, sex, body mass index (BMI), and worst-case/best-case deterministic imputation (Supplementary Tables 2 and 3).

#### Secondary and Exploratory Outcomes

All secondary outcomes are exploratory; no multiplicity adjustment was performed. All secondary continuous outcomes were non-normally distributed (Shapiro-Wilk; all p < 0.05; Supplementary Table 4) and analyzed using Kruskal-Wallis with Mann-Whitney U post hoc comparisons. Categorical variables used Fisher's exact test (p < 0.05). Analyses were performed using SPSS 18.0 and Stata (multiple imputation). Secondary outcomes were analyzed cross-sectionally at each timepoint rather than with a repeated-measures model; this approach does not account for within-subject correlation across visits and is considered acceptable given the exploratory, hypothesis-generating nature of these outcomes.

## Results

Of 216 enrolled Thai participants, 8 (3.7%) were lost to follow-up and included in the primary ITT analysis through multiple imputation. Among 208 completers, 90.9% were female, mean age 61.7 ± 7.1 years, mean BMI 26.2 ± 4.5 kg/m^2^, and 73.6% had Kellgren-Lawrence grade 2 OA. Baseline characteristics are presented in Table I.

**Table I:** Demographic Data of All Treatment Groups

Characteristics	UC-II (N = 51)	pCGS (N = 52)	Diacerein (N = 52)	Placebo (N = 53)
Female (n, %)	48 (94.1%)	50 (96.2%)	44 (84.6%)	47 (88.7%)
Age (years) mean ± SD	61.2 ± 6.0	61.2 ± 6.7	61.4 ± 8.2	63.2 ± 7.4
BMI (kg/m^2^) mean ± SD	27.3 ± 4.7	26.5 ± 4.1	25.5 ± 4.7	25.5 ± 4.5
Kellgren-Lawrence grade (n, %)				
Grade 2	35 (68.6%)	43 (82.7%)	37 (71.2%)	38 (71.2%)
Grade 3	16 (31.4%)	9 (17.3%)	15 (28.8%)	15 (28.7%)
Flexion (°) mean ± SD	121.4 ± 10.1	122.4 ± 10.8	123.3 ± 8.0	122.5 ± 10.3
Extension (°) mean ± SD	1.3 ± 3.1	0.4 ± 1.7	0.8 ± 1.8	0.9 ± 3.3
Underlying disease (n, %)				
Hypertension	25 (49.0%)	20 (38.5%)	15 (28.8%)	21 (39.6%)
Diabetes mellitus	9 (17.6%)	8 (15.4%)	10 (19.2%)	10 (18.9%)
Dyslipidemia	15 (29.4%)	15 (28.8%)	12 (23.1%)	15 (28.3%)
Asthma	2 (3.9%)	0 (0%)	3 (5.8%)	0 (0%)
Hypothyroid	1 (2.0%)	1 (1.9%)	0 (0%)	0 (0%)
Hyperthyroid	0 (0%)	0 (0%)	0 (0%)	2 (3.8%)
Migraine	1 (2.0%)	1 (1.9%)	1 (1.9%)	2 (3.8%)
Gouty arthritis	1 (2.0%)	0 (0%)	1 (1.9%)	0 (0%)

Extension (°) values represent the flexion contracture (degrees short of full extension); higher values indicate greater extension deficit.

BMI = body mass index, pCGS = prescription-grade crystalline glucosamine sulfate (Viatryl-S), and UC-II = undenatured type II collagen.

No statistically significant between-group differences in baseline characteristics (all p > 0.05).

### Primary Outcome (WOMAC Total Score at Week 24)

All groups demonstrated substantial WOMAC improvement within the first 12 weeks, including placebo, reflecting a prominent placebo effect from standardized conservative care (Table II). In the primary ITT analysis (Table III), estimated marginal means at Week 24 were the following: UC-II 42.3 (95% CI 32.4-52.3), pCGS 39.0 (29.3-48.7), diacerein 34.7 (25.0-44.4), and placebo 47.0 (37.4-56.7). Between-group differences vs. placebo were as follows: UC-II −4.7 (−18.6 to 9.1), pCGS −8.1 (−21.8 to 5.7), and diacerein −12.3 (−25.9 to 1.3) (Table III). All 95% CIs included zero, and all point estimates were at or below, and none clearly exceeded the minimum clinically important difference threshold of 12.5 points^28^, indicating no statistically or clinically meaningful superiority over placebo. Week 36 results were consistent, with differences of −1.3 to −4.8 points (Table III)

**Table II:** The Primary Outcome Comparison of All Treatment Groups (Mean ± SD, Per-Protocol Analysis)

Variable	UC-II (N = 51)	pCGS (N = 52)	Diacerein (N = 52)	Placebo (N = 53)	p
WOMAC-pain					
Baseline	19.76 (9.92)	18.25 (7.95)	21.63 (10.41)	21.78 (9.75)	—
Week 12	8.92 (7.78)	11.02 (9.82)	10.37 (10.13)	9.92 (8.82)	0.813
Week 24*	9.08 (8.5)	9.77 (9.61)	9.21 (9.9)	10.98 (11)	0.866
Week 36	9 (8.45)	9.62 (9.63)	10.13 (10.20)	8.49 (8.08)	0.967
WOMAC-stiffness					
Baseline	6.82 (4.18)	5.25 (4.61)	6.57 (5.05)	7.89 (4.62)	—
Week 12	3.73 (4.29)	4.19 (4.06)	3.75 (4.2)	4.23 (3.62)	0.522
Week 24*	3.94 (3.71)	4.21 (4.13)	3.6 (4.17)	5.53 (4.48)	0.075
Week 36	3.71 (3.43)	3.94 (3.71)	4.29 (4.43)	4.72 (3.59)	0.307
WOMAC-function					
Baseline	51.14 (31.47)	37.25 (21.4)	51 (31.24)	54.81 (31.13)	—
Week 12	25.80 (23.64)	25.95 (21.31)	24.98 (23.45)	27 (23.32)	0.928
Week 24*	29.29 (24.51)	25.5 (23.28)	22.5 (25.13)	31.21 (28.5)	0.207
Week 36	30.18 (26.51)	25.77 (22.06)	28.83 (27.69)	30.89 (29.97)	0.852
WOMAC-total					
Baseline	77.73 (41.78)	60.74 (29.44)	79.2 (42.21)	84.48 (41.57)	—
Week 12	38.45 (32.93)	42.35 (31.93)	39.1 (35.11)	41.23 (34.25)	0.850
Week 24*	42.31 (32.32)	38.74 (34.51)	34 (36.94)	46.83 (39.46)	0.135
Week 36	42.88 (35.04)	39.33 (33.27)	43.25 (39.2)	44.09 (36.51)	0.924

Values are mean (SD). p-values from Kruskal-Wallis test (non-normally distributed outcomes).

— = baseline comparison not performed (prerandomization), pCGS = prescription-grade crystalline glucosamine sulfate (Viatryl-S), UC-II = undenatured type II collagen, and WOMAC = Western Ontario and McMaster Universities Arthritis Index.

* Primary timepoint (Week 24, end of treatment). Week 36 = post-treatment follow-up (secondary timepoint).

**Table III:** WOMAC Scores — Estimated Marginal Means and Between-group Differences vs. Placebo (ITT Analysis, MICE + LME, N = 216)

	Estimated Marginal Means (95% CI)				Difference vs. Placebo (95% CI)		
Variable	UC-II (N = 51)	pCGS (N = 52)	Diacerein (N = 52)	Placebo (N = 53)	UC-II vs. Placebo	pCGS vs. Placebo	Diacerein vs. Placebo
WOMAC-pain							
Week 0	19.3 (16.8, 21.8)	18.4 (15.9, 20.9)	21.6 (19.1, 24.1)	21.8 (19.3, 24.3)	−2.5 (−6.0, 1.0)	−3.4 (−6.9, 0.1)	−0.1 (−3.7, 3.4)
Week 12	8.8 (6.2, 11.4)	10.8 (8.2, 13.3)	10.2 (7.7, 12.8)	9.8 (7.3, 12.3)	−1.0 (−4.6, 2.6)	1.0 (−2.6, 4.5)	0.4 (−3.2, 4.0)
Week 24†	9.0 (6.4, 11.6)	9.6 (7.1, 12.2)	9.1 (6.6, 11.6)	10.8 (8.3, 13.3)	−1.8 (−5.4, 1.8)	−1.1 (−4.7, 2.5)	−1.7 (−5.3, 1.9)
Week 36	8.7 (6.1, 11.2)	9.4 (6.9, 11.9)	9.8 (7.3, 12.4)	8.3 (5.8, 10.8)	0.4 (−3.2, 4.0)	1.1 (−2.5, 4.7)	1.6 (−2.0, 5.2)
WOMAC-stiffness							
Week 0	6.8 (5.7, 7.9)	5.2 (4.1, 6.3)	6.6 (5.5, 7.7)	7.9 (6.8, 9.0)	−1.1 (−2.6, 0.5)	−2.6 (−4.2, −1.1)*	−1.3 (−2.9, 0.2)
Week 12	3.6 (2.5, 4.8)	4.0 (2.9, 5.1)	3.7 (2.6, 4.8)	4.2 (3.1, 5.3)	−0.5 (−2.1, 1.1)	−0.2 (−1.8, 1.4)	−0.5 (−2.1, 1.1)
Week 24†	4.0 (2.8, 5.1)	4.1 (3.0, 5.2)	3.5 (2.5, 4.7)	5.5 (4.4, 6.6)	−1.5 (−3.1, 0.0)	−1.4 (−2.9, 0.2)	−1.9 (−3.5, −0.4)*
Week 36	3.5 (2.4, 4.6)	3.8 (2.7, 4.9)	4.1 (3.0, 5.2)	4.6 (3.5, 5.7)	−1.1 (−2.7, 0.4)	−0.8 (−2.4, 0.7)	−0.5 (−2.0, 1.1)
WOMAC-function							
Week 0	49.8 (42.9, 56.7)	37.1 (30.3, 44.0)	51.0 (44.1, 57.9)	54.8 (47.9, 61.7)	−5.0 (−14.8, 4.7)	−17.7 (−27.4, −7.9)*	−3.8 (−13.6, 5.9)
Week 12	25.4 (18.3, 32.5)	25.4 (18.4, 32.3)	24.5 (17.5, 31.5)	26.6 (19.7, 33.6)	−1.3 (−11.2, 8.7)	−1.3 (−11.1, 8.5)	−2.1 (−11.9, 7.7)
Week 24†	29.3 (22.2, 36.5)	25.2 (18.2, 32.2)	22.1 (15.1, 29.1)	30.8 (23.8, 37.8)	−1.4 (−11.5, 8.6)	−5.6 (−15.5, 4.3)	−8.7 (−18.5, 1.1)
Week 36	29.3 (22.3, 36.4)	25.2 (18.3, 32.2)	28.0 (21.0, 34.9)	30.3 (23.4, 37.3)	−1.0 (−11.0, 9.0)	−5.1 (−14.9, 4.7)	−2.4 (−12.2, 7.4)
WOMAC-total							
Week 0	75.8 (66.3, 85.4)	60.7 (51.1, 70.3)	79.1 (69.6, 88.7)	84.5 (74.9, 94.0)	−8.6 (−22.1, 4.9)	−23.8 (−37.3, −10.3)*	−5.3 (−18.9, 8.2)
Week 12	37.8 (27.9, 47.8)	40.1 (30.5, 49.8)	38.4 (28.7, 48.2)	40.6 (31.0, 50.2)	−2.8 (−16.6, 11.0)	−0.5 (−14.1, 13.1)	−2.2 (−15.8, 11.5)
Week 24†	42.3 (32.4, 52.3)	39.0 (29.3, 48.7)	34.7 (25.0, 44.4)	47.0 (37.4, 56.7)	−4.7 (−18.6, 9.1)	−8.1 (−21.8, 5.7)	−12.3 (−25.9, 1.3)
Week 36	41.5 (31.8, 51.3)	38.4 (28.8, 48.0)	42.0 (32.3, 51.6)	43.3 (33.7, 52.9)	−1.8 (−15.5, 12.0)	−4.8 (−18.4, 8.7)	−1.3 (−14.9, 12.3)

Values are estimated marginal means (95% CI) from linear mixed-effects models with MICE (m = 10), ITT analysis (N = 216); and mean differences vs. placebo (95% CI) from the same model.

ITT = intention to treat, LME = linear mixed-effects, MICE = multiple imputation by chained equations, pCGS = prescription-grade crystalline glucosamine sulfate (Viatryl-S), UC-II = undenatured type II collagen, and WOMAC = Western Ontario and McMaster Universities Arthritis Index.

* Indicates 95% CI excludes zero (statistically significant difference vs. placebo).

† Primary timepoint (Week 24, end of treatment). Week 36 = post-treatment follow-up (secondary timepoint).

Baseline significant differences in pCGS (Week 0) reflect randomization imbalance, not treatment effect; all postbaseline comparisons adjusted for baseline WOMAC covariate.

### Secondary and Exploratory Outcomes

No significant between-group differences were observed for NPRS, JSW, TUG, 6MWT, or rescue analgesic use at any timepoint (Table IV). JSW findings should be interpreted in the context of the MDC_95_ of 1.22 mm and short study duration; all secondary outcomes are considered exploratory.

**Table IV:** Secondary and Exploratory Outcomes — All Treatment Groups (Mean ± SD or Median [IQR])

Variable	UC-II (N = 51)	pCGS (N = 52)	Diacerein (N = 52)	Placebo (N = 53)	p
NPRS; mean (SD)					
Baseline	5.98 (1.33)	5.68 (1.27)	6.33 (1.53)	5.94 (1.49)	—
Week 12	3.57 (2.32)	4.25 (2.04)	3.48 (2.29)	3.47 (1.98)	0.203
Week 24	3.27 (2.43)	3.63 (2.34)	3.15 (2.28)	3.45 (2.28)	0.842
Week 36	2.94 (2.2)	3.13 (2.24)	2.79 (2.4)	2.83 (2.15)	0.823
JSW; mean (SD)					
Baseline	3.23 (1.2)	3.61 (1.25)	3.61 (1.3)	3.58 (1.33)	—
Week 12	3.02 (1.13)	3.38 (1.16)	3.30 (1.21)	3.36 (1.25)	0.381
Week 24	2.95 (1.13)	3.17 (1.17)	3.15 (1.18)	3.33 (1.28)	0.502
Week 36	2.9 (1.21)	3.23 (1.27)	3.17 (1.26)	3.27 (1.27)	0.439
6MWT; mean (SD)					
Baseline	206.24 (50.96)	220.15 (47.61)	205.63 (60.46)	198.31 (44.28)	—
Week 24	259.54 (68.83)	255.6 (69.52)	261.73 (73.81)	248.86 (61.13)	0.786
Week 36	267.64 (64.48)	252.87 (58.19)	260.75 (69.55)	242.89 (60.88)	0.226
TUG; mean (SD)					
Baseline	13.89 (4.9)	12.63 (3.56)	13.13 (4.03)	13.84 (4.58)	—
Week 24	11.83 (3.41)	11.46 (2.8)	11.47 (2.82)	11.64 (3.23)	0.985
Week 36	11.26 (3.52)	11.35 (2.51)	11.91 (3.97)	11.97 (3.53)	0.648
Paracetamol; median (IQR)					
Week 4	0 (0-3)	0 (0-1.5)	0 (0-2)	0 (0-1.5)	0.742
Week 12	0 (0-1.5)	0 (0-1)	0 (0-5)	0 (0-5)	0.694
Week 24	0 (0-0)	0 (0-8.75)	0 (0-0)	0 (0-2.5)	0.656
Week 36	0 (0-0)	0 (0-0)	0 (0-10)	0 (0-2.5)	0.322
Naproxen; median (IQR)					
Week 4	0 (0-5)	0.5 (0-9.25)	0 (0-4.25)	0 (0-5)	0.557
Week 12	0 (0-5)	0 (0-10.50)	0 (0-10)	0 (0-7)	0.827
Week 24	0 (0-5)	4 (0-10)	0 (0-10)	0 (0-15.25)	0.387
Week 36	0 (0-15)	3 (0-20)	2.5 (0-17.5)	0 (0-15.25)	0.909

Values are mean (SD) unless otherwise stated: p-values from Kruskal-Wallis test (continuous variables) or Fisher's exact test (categorical variables). All secondary outcomes are exploratory; no multiplicity adjustment was performed. 6MWT and TUG not assessed at Week 12 (safety visit only).

6MWT = 6-minute walk test (m), IQR = interquartile range, JSW = joint space width (mm), NPRS = Numeric Pain Rating Scale (0-10), and TUG = timed up and go (seconds).

## Discussion

In this 4-arm, double-blind, placebo-controlled randomized clinical trial (RCT), none of the 3 supplements (UC-II, pCGS, or diacerein) demonstrated superior efficacy over placebo in WOMAC score or any secondary outcome including NPRS, JSW, 6MWT, TUG, analgesic use, or adverse events over 36 weeks. The observed improvements across all groups, including the placebo arm, likely reflect the standardized conservative care provided uniformly to all participants, emphasizing self-management, lifestyle modification, and exercise.

To our knowledge, this is the first RCT comparing these 3 supplements against placebo. Our findings align with the Glucosamine/Chondroitin Intervention Trial (no significant pain reduction with glucosamine or chondroitin vs. placebo)^29^, diacerein trials showing no benefit over NSAIDs alone^30^, and Cochrane reviews finding insufficient evidence for either^31^ or diacerein^32^ in knee OA

Contrasting evidence exists, though with methodological limitations: Meta-analyses suggest structural benefits with long-term glucosamine (1,500 mg/day)^33^, and a 3-year RCT demonstrated modest symptom improvements^34^, suggesting 24 weeks may be insufficient to capture full benefit. Brahmachari et al.^35^ reported diacerein reduced pain in early knee OA, but the single-blinded 12-week design, small sample (n = 55), and high (31%) dropout limit comparability.

The efficacy of UC-II remains controversial. Yuenyongviwat et al.^36^ found no superiority of combined UC-II and hydrolyzed collagen over placebo at 12 weeks. Sadigursky et al.^37^ reported benefits with UC-II at 90 days but lacked a placebo control and blinded evaluators. Kumar et al.'s review^16^ suggested early benefit but was limited by high heterogeneity (*I*^2^ = 70%), small numbers (n = 243), and inconsistent dosing. Taken together, positive findings in the literature are possibly related to methodological limitations, heterogeneity, and follow-up periods, rather than true supplement efficacy.

All 3 supplements demonstrated excellent safety profiles consistent with prior literature^31,32^. Diacerein was associated with minor gastrointestinal effects such as diarrhea^38^, though the European Medicines Agency has affirmed its safety^39^. UC-II is FDA registered as a dietary ingredient and generally recognized as safe^17^. Our findings confirm comparable safety across all groups, with no serious adverse events or withdrawals (Supplementary Table 5).

The management of mild-to-moderate OA presents challenges due to inconsistent supplement guidelines across major medical organizations. While the European Society for Clinical and Economic Aspects of Osteoporosis, Osteoarthritis, and Musculoskeletal Diseases (ESCEO) 2019 guideline recommends glucosamine sulfate and diacerein^2^, the ACR 2019 guideline specifically discourages their use^11^. The OA Research Society International 2019 guideline offers no supplement recommendations^13^, and the American Academy of Orthopedic Surgeons 2021 guideline notes inconsistent evidence regarding glucosamine, calling for further research^12^. Most recently, the National Institute for Health and Care Excellence 2022 guideline explicitly recommends against glucosamine for knee OA patients^40^. Notably, UC-II is absent from all these guidelines, highlighting a significant knowledge gap.

Our findings clarify the clinical utility of knee OA supplements and suggest that clinicians should prioritize conservative strategies such as education, lifestyle modification, and exercise over the routine use of UC-II, glucosamine sulfate, or diacerein. Given the lack of superior efficacy observed in this study, these results should inform the development of evidence-based guidelines and healthcare reimbursement policies. Ultimately, larger standardized trials with extended follow-up periods are required before these supplements can be confidently recommended for the management of mild-to-moderate knee OA.

### Limitations

This study has several limitations. First, the 24-week treatment duration may be insufficient to detect full supplement benefits, particularly for structural outcomes; longer trials are needed. Second, 8 participants (3.7%) were lost to follow-up, though ITT analysis with multiple imputation mitigates this concern. Secondary outcomes were analyzed timepoint-by-timepoint rather than with a repeated-measures approach, which does not account for within-subject correlation over time; this is considered acceptable given their exploratory, hypothesis-generating role. Third, exclusion of recent intra-articular injection recipients may limit generalizability to real-world practice where supplements are often used alongside other interventions. Fourth, JSW assessment over 24 to 36 weeks is insufficient to detect meaningful structural change and should be interpreted as exploratory. Fifth, this single-center Thai population study may limit generalizability to other ethnic groups and healthcare settings. Finally, serum biomarkers of cartilage metabolism were not measured, precluding insight into biological mechanisms^41^.

## Conclusions

In conclusion, UC-II, pCGS, and diacerein did not demonstrate superiority over placebo in reducing WOMAC score at the primary end point of 24 weeks, with consistent results through 36 weeks, in patients with mild-to-moderate knee OA. Despite their favorable safety profiles, our findings do not support the routine use of these supplements as monotherapy, and standard conservative management should remain the primary treatment strategy.

## Funding

This research project is supported by the Faculty of Medicine Siriraj Hospital, Mahidol University, the Medical Association of Thailand and Government Pharmaceutical Organization.

## Data Availability

Anonymized data will be shared on reasonable request.

## Appendix

Supporting material provided by the authors is posted with the online version of this article as a data supplement at jbjs.org (https://links.lww.com/JBJSOA/B328). This content was not copyedited or verified by JBJS.
